# Adequacy of gestational weight gain according to the brazilian charts: a comparison with the Institute of Medicine recommendations

**DOI:** 10.61622/rbgo/2026rbgo99

**Published:** 2026-02-20

**Authors:** Mariana Campos de Moraes, Luciana da Cunha Bernardes Argenta, Sanmira Lopes Fagherazzi, Gabriella Pinto Belfort, Beatriz Magalhães Blois dos Santos, Laura Lima Camelo, Letícia Barbosa Gabriel da Silva, Claudia Saunders

**Affiliations:** 1 Maternidade Escola da Universidade Federal do Rio de Janeiro Rio de Janeiro RJ Brazil Maternidade Escola da Universidade Federal do Rio de Janeiro, Rio de Janeiro, RJ, Brazil.; 2 Universidade Federal do Estado do Rio de Janeiro Escola de Nutrição Rio de Janeiro RJ Brazil Escola de Nutrição, Universidade Federal do Estado do Rio de Janeiro, Rio de Janeiro, RJ, Brazil.; 3 Universidade Federal do Rio de Janeiro Instituto de Nutrição Josué de Castro Rio de Janeiro RJ Brazil Instituto de Nutrição Josué de Castro, Universidade Federal do Rio de Janeiro, Rio de Janeiro, RJ, Brazil.

**Keywords:** Gestational weight gain, Prenatal nutrition, Prenatal care, Pregnancy, Pregnant people

## Abstract

**Objective:**

To analyze the adequacy of gestational weight gain (GWG) and associated factors, according to the Brazilian GWG charts, and compare with the Institute of Medicine (IOM) recommendations.

**Methods:**

This was a cross-sectional study. Sociodemographic, clinical, and obstetric data were collected from interviews and medical records. The sample was divided into three groups according to the Brazilian charts: insufficient GWG, adequate GWG, and excessive GWG. The chi-square and Kruskal-Wallis tests were used to compare frequencies and medians, respectively, and Dunn's test was used to verify differences between groups, assuming statistical significance of p<0.05.

**Results:**

Seven hundred and twenty-five women with low-and high-risk pregnancies were evaluated. Using the Brazilian charts, the prevalence of insufficient, adequate, and excessive GWG was 25.8%, 21.1%, and 53.1%, respectively. Compared with the classifications given using the IOM method, there was a lower prevalence of insufficient and adequate GWG and a higher prevalence of excessive GWG. The sensitivity and specificity of the Brazilian charts versus the IOM recommendations were 100% and 76.6%, respectively, for excessive GWG and 72.3% and 20%, respectively, for insufficient GWG. The modifiable factors related to GWG were pregestational BMI (p=0.001) and number of consultations with a nutritionist (p=0.008).

**Conclusion:**

Studies evaluating the Brazilian GWG charts are scarce. The Brazilian charts indicated a higher prevalence of excessive GWG than the IOM recommendations. The sensitivity and specificity of the Brazilian charts were better for excessive GWG than for insufficient GWG, using the IOM recommendations as the standard.

## Introduction

Weight gain is a normal and physiological aspect of human pregnancy, due to fetal-placental growth and an increase in maternal tissues, especially adipose tissue.^(1)^ The Brazilian Ministry of Health considers gestational weight gain (GWG) monitoring an important part of prenatal care, since its influence on the occurrence of adverse perinatal outcomes has been recognized since 1970, when the American report *Maternal Nutrition and the Course of Pregnancy* was published.^(2,3)^

Insufficient GWG is associated with a higher risk of low birth weight (birth weight < 2500 g), small-for-gestational-age newborns, preterm birth, and microcephaly.^(4)^ Meanwhile, excessive GWG is associated with cesarean sections, gestational diabetes mellitus (GDM), hypertensive disorders of pregnancy (HDP), large-for-gestational-age (LGA) newborns, macrosomia (birth weight ≥ 4000 g), preterm birth, and longer-term outcomes in offspring, such as childhood obesity.^(4,5)^

According to Carrilho et al.,^(6)^ in the past few years, there has been an increase in insufficient GWG among Brazilian women and an increase in maternal pregestational overweight and obesity rates, considered a predictor of GWG.^(3,6)^ These data reinforce the need for continuous monitoring of weight gain during pregnancy.

Until 2022, the Brazilian Ministry of Health recommended two methods for GWG monitoring. The first, developed by the United States Institute of Medicine (IOM), uses body mass index (BMI) to diagnose women's pregestational nutritional status and thus recommend the appropriate weight gain range.^(3,7)^ The second, developed by Atalah et al.,^(8)^ considers gestational BMI and gestational age (GA) at the time of assessment, and was used mainly when the pregestational BMI was unknown.^(8,2)^ The World Health Organization (WHO),^(9)^ in its publication *WHO Recommendations on Antenatal Care for a Positive Pregnancy Experience*, states that the ideal GWG varies according to population characteristics, but recommends the ranges established by the IOM.

Despite their ease of use, both recommendations have limitations. The table published by Atalah et al. ^(8)^ does not predict the occurrence of insufficient birth weight, has low sensitivity and specificity for the BMI cutoff points, and overestimates underweight in Brazilian pregnant women.^(10)^ Martínez-Hortelano et al.^(11)^ suggest that the IOM recommendations tend to continually increase the incidence of overweight and obese among women of reproductive age and that interventions in GWG should be adapted regionally. Both the Atalah et al.^(8)^ and the IOM recommendations were developed using data from populations with characteristics vastly different from those of the Brazilian female population.^(12)^

In view of the above, the Brazilian Maternal and Child Nutrition Consortium created new GWG recommendations, published in its Brazilian Gestational Weight Gain Charts.^(12)^ To develop these charts, it used data from 17,344 healthy adult Brazilian women with a singleton pregnancy.^(13)^ The Ministry of Health first adopted these charts for monitoring GWG in prenatal care in 2022, when it published the *Caderneta da Gestante* (providing advice and a record of follow-up for women during and after pregnancy), which it updated in 2024, and with the incorporation of these recommendations in the primary care food and nutrition surveillance guide.^(14,15)^

This study aimed to analyze the adequacy and associated factors of GWG among women receiving care at a public maternity hospital in Rio de Janeiro, Brazil, according to the Brazilian GWG charts, and compare them with IOM recommendations.

## Methods

This was an observational cross-sectional study with a quantitative approach. Data were used from a database constructed as part of a pre-existing study, which was approved by the research ethics committee of the Maternity School of the Federal University of Rio de Janeiro (Decision nº 6.783.293, CAAE: 46338021.4.0000.5275), preserving the anonymity of the participants.

Data were collected between 2021 and 2023 in face-to-face interviews with adult women who were hospitalized in wards immediately after giving birth and by consulting their medical records. The hospital in question was a public maternity hospital in the city of Rio de Janeiro, Brazil, and the data were collected by researchers from the Maternal and Infant Health Research Group (Grupo de Pesquisa em Saúde Materna e Infantil) after receiving training for this purpose. Sociodemographic data, current and previous clinical and obstetric history, and data on prenatal obstetric and nutritional care were used.

The following inclusion criteria were adopted: maternal age at conception ≥ 18 years; at least one prenatal consultation in any health facility; singleton pregnancy; and availability of anthropometric data in medical records (height, pregestational weight, and pre-delivery weight).

To assess the adequacy of GWG, the Brazilian weight gain charts^(15)^ and the IOM gestational weight gain recommendations were used. Both use pregestational BMI as a parameter to define the ideal weight gain range for each woman. Pregestational BMI was classified using the WHO method, namely, pregestational weight (kg) divided by the woman's height squared.^(7)^ Pregestational weight was recorded as either the women's self-reported weight up to two months before conception,^(16)^ their usual weight, or the weight measured up to the 8th gestational week.^(17)^ Total GWG was calculated as the difference between pregestational weight and pre-delivery weight.^(15)^

GA at delivery was based on the ultrasound exam in the first trimester. In the absence of this, GA was calculated according to the date of the last menstrual period. In cases of full-term pregnancies, GWG ranges were recommended according to pregestational BMI based on the Brazilian charts (Table 1) and IOM (Table 2), and GWG was then classified as insufficient, adequate, or excessive.

**Table 1:** Gestational weight gain (GWG) recommendation ranges, according to pregestational body mass index (BMI), by Brazilian weight gain charts

Pre-pregnancy BMI (kg/m^2^)	Classification of pre-pregnancy BMI	GWG recommendation ranges (kg)
< 18.5	Low weight	9.7–12.2
≥18.5 and < 25	Normal weight	8.0–12.0
≥ 25 and < 30	Overweight	7.0–9.0
≥ 30	Obesity	5.0–7.2

**Source:** Adapted from Ministério da Saúde.^15^

**Table 2:** Gestational weight gain (GWG) recommendation ranges, according to pregestational body mass index (BMI), by Institute of Medicine

Pre-pregnancy BMI (kg/m^2^)	Classification of prepregnancy BMI	Weekly recommended GWG (kg) in the 2nd and 3rd trimesters	GWG recommendation ranges (kg)
< 18.5	Low weight	0.5 (0.44–0.58)	12.5 – 18.0
≥18.5 and < 25	Normal weight	0.4 (0.35–0.50)	11.5 – 16.0
≥ 25 and < 30	Overweight	0.3 (0.23–0.33)	7.0 – 11.5
≥ 30	Obesity	0.2 (0.17–0.27)	5.0 – 9.0

**Source:** Adapted from IOM.^3^

To verify the adequacy of the Brazilian charts for preterm births, the total GWG that corresponded to the woman's pregestational nutrition status was calculated for the GA at delivery.^(15)^ To assess the adequacy of preterm pregnancies according to the IOM, the number of weeks at delivery was multiplied by the corresponding recommended GWG range (Table 2), thus defining the minimum and maximum recommended for that GA.

The participants were classified according to the adequacy of their GWG by the Brazilian charts, they were divided into three groups: adequate, insufficient, or excessive GWG. These data were analyzed in relation to their sociodemographic, clinical, and obstetric characteristics to identify the factors associated with GWG adequacy.

Aiming for equality in care, at the Brazilian public health system, medium- and high-risk pregnancies are assisted in specialized units, including ones with previous diabetes, GDM, HDP and other health conditions, while low-risk pregnancies stay at primary prenatal care.^(2)^ For this reason, in this study, women who received secondary or tertiary prenatal care were defined as having medium/high-risk pregnancies; those who received only primary prenatal care were classified as having low-risk pregnancies.

IBM SPSS^®^ Statistics 31 was used for data analysis. Quantitative variables were described as medians and interquartile ranges (IQR). Data normality was evaluated using the Shapiro-Wilk test and graphical visualization. Categorical variables were described as absolute and relative frequencies. In the statistical analysis, the Kruskal-Wallis test was used to compare medians, Dunn's post-hoc test was used to verify differences between medians, and Pearson's chi-squared test was used to compare frequencies. The bivariate Pearson correlation test was used to assess the correlation between obstetric variables and total GWG. Statistical significance was set at p<0.05. The sensitivity and specificity of the insufficient and excessive GWG classifications were calculated for the Brazilian charts in relation to the values given by the IOM.^(3)^

## Results

The study enrolled 725 adult women, whose median age at conception was 27.0 (23.0– 33.0) years. Of this total, 47.6% (n=345) lived in the south zone of Rio de Janeiro, and 92.2% (n=666) said they had adequate sanitation conditions in their homes, with regular garbage collection, running water, and connection to the sewage system. The self-identified skin color of 69.9% (n=506) was non-white, 71.1% (n=209) had completed high school, 61.1% (n=441) had paid work, and 82.1% (n=587) lived with a partner. The median number of people per household was 4.0 (3.0–5.0), and the median household per capita income was 0.51 (0.33–0.82) times the minimum wage (Table 3).

**Table 3:** Sociodemographic profile of women receiving postpartum care at the Maternity School of the Federal University of Rio de Janeiro, classified by gestational weight gain calculated using Brazilian charts

Classification of total GWG according to brazilian weight gain charts						
Sociodemographic characteristics		Median (IQR)	Insufficient n=187	Adequate n=153	Excessive n=385	p-value*
			Median (IQR)	Median (IQR)	Median (IQR)	
Age at conception (years, n=725)		27.0 (23.0–33.0)	29.0 (25.0–34.0) ^a^	27.0 (23.0-32) a,b	27.0 (23.0–32.0)^b^	0.01
		**n(%)**	**n(%)**	**n(%)**	**n(%)**	p-value**
Self-reported skin color (n=724)						
	White	218(30.1)	58(26.6)	48(22.0)	112(51.4)	0.82
	Non-white	506(69.9)	128(25.3)	105(20.8)	273(54.0)	
Instruction level (n=724)						
	Incomplete high school	209(28.9)	60(28.7)	32(15.3)	117(56.0)	0.05
	High school graduate	515(71.1)	127(24.7)	121(23.5)	267(51.8)	
Place of residence (n=725)						
	South zone of Rio de Janeiro	345(47.6)	95(27.5)	75(21.7)	175(50.7)	0.64
	North zone of Rio de Janeiro	174(24.0)	38(21.8)	37(21.3)	99(56.9)	
	Others regions	206(28.4)	54(26.2)	41(19.9)	111(53.9)	
Sanitation conditions (n=722)						
	Adequate	666(92.2)	174(26.1)	137(20.6)	355(53.3)	0.63
	Inadequate	56(7.8)	12(21.4)	14(25.0)	30(53.6)	

* Kruskal-Wallis test /

** Chi-square test /

a,b,c Dunn's post-hoc test; medians followed by different letters are significantly different from each other.

Note: GWG = gestational weight gain; IQR = interquartile range.

As for the pregnancies, 64.7% (n=469) were classified as low risk and 35.3% (n=256), as medium/high risk. Regarding the women's clinical and obstetric history, the median numbers of previous pregnancies, births, and miscarriages were 2.0 (1–3), 2.0 (1–3), and 0 (0–1), respectively, with the median interpregnancy interval being 48.5 (17.25–85) months. Total median GWG was 10.5 (6–14.3) kg, the median number of prenatal consultations was 9.0 (8–11), and the median number of nutritional prenatal consultations was 0.0 (0– 2).

Table 3 presents the sociodemographic, clinical, and obstetric characteristics of the participants, associated with GWG adequacy classifications. The women with insufficient GWG had a higher median age at conception (29 years) than those who had excessive GWG (27 years, p=0.01).

Table 4 shows the participants’ clinical and obstetric characteristics grouped by the GWG adequacy classifications using the Brazilian charts. The number of previous deliveries was associated with GWG (p=0.02) and the IQR was higher among insufficient GWG group (1-3) than among adequate (1-2) and excessive (1-2) ones. In the same way, the number of previous pregnancies appears to influence GWG (p=0.02). A negative correlation was found between the number of pregnancies (r = −0.11, p < 0.001) and births (r = −0.15, p < 0.001) and total GWG (data not shown in tables). That is, as the number of pregnancies and births increases, gestational weight gain decreases.

**Table 4:** Clinical and obstetric characteristics of women receiving postpartum care at the Maternity School of the Federal University of Rio de Janeiro, classified by gestational weight calculated using Brazilian charts

Classification of total GWG according to Brazilian weight gain charts						
			Insufficient n=187	Adequate n=153	Excessive n=385	
Clinical and obstetric characteristics		Median (IQR)	Median (IQR)	Median (IQR)	Median (IQR)	p-value*
Obstetric history (n=725)						
	Number of previous pregnancies	2.0 (1.0–3.0)	2.0 (1.0–3.0) a	2.0 (1.0–3.0) b	2.0 (1.0–3.0) b	0.02
	Number of previous deliveries	2.0 (1.0–3.0)	2.0 (1.0–3.0) a	2.0 (1.0–2.0) b	2.0 (1.0–2.0) b	0.02
	Number of previous miscarriages	0.0 (0.0–1.0)	0.0 (0.0–1.0)	0.0 (0.0–1.0)	0.0 (0.0–1.0)	0.71
	Gestational age at first prenatal consultation (weeks, n=525)	8.1 (6.4–11.4)	8.3 (6.3–11.6)	8.1 (6.4–11.4)	8.1 (6.6–11.4)	0.87
	Total GWG (kg, n=725)	10.5 (6.0–14.3)	3.7 (1.4– 5.8) a	8.8 (6.9–8.8) b	14.0 (11.3–17.6) c	<0.001
	Number of prenatal consultations (n=718)	9.0 (8.0–11.0)	10.0 (8.0–11.0)	9.0 (8.0–10.0)	9.0 (8.0–11.0)	0.26
	Number of consultations with nutritionists (n=716)	0.0 (0.0–2.0)	0.0 (0.0–3.0) a	0.0 (0.0–2.0) b	0.0 (0.0–2.0) b	0.01
		**n(%)**	**n(%)**	**n(%)**	**n(%)**	p-value**
Total GWG according to IOM^3^ (n=725)						
	Insufficient	244(33.6)	187(6.6)	57(23.4)	0(0)	<0.001
	Adequate	226(31.2)	0(0)	9(42.5)	130(57.5)	
	Excessive	255(35.2)	0(0)	0(0)	255(100)	
Pregnancy risk (n=725)						
	Medium/high risk	344(47.4)	102(54.5)	64(41.8)	178(46.2)	0.05
	Low risk	381(52.6)	85(45.5)	89(58.2)	207(53.8)	
Pre-pregnancy BMI classification (n=725)						
	Low weight	32(4.4)	5(15.6)	8(25.0)	19(59.4)	<0.001
	Normal weight	246(33.9)	51(20.7)	70(28.5)	125(50.8)	
	Overweight	215(29.7)	56(26.0)	30(14.0)	129(60.0)	
	Obesity	232(32.0)	75(32.3)	45(19.4)	112(48.3)	
Presence of chronic disease (n=719)						
	Yes	190(26.4)	53(27.9)	34(17.9)	103(54.2)	0.39
	No	529(73.6)	131(24.8)	118(22.3)	280(52.9)	
Maternal complications (n=719)						
	None	307(42.7)	65(21.2)	72(23.5)	170(55.4)	<0.001
	HDP	90(12.5)	14(15.6)	18(20.0)	58(64.4)	
	GDM	131(18.2)	59(45.0)	18(13.7)	54(41.2)	
	HDP and GDM	34(4.7)	7(20.6)	8(23.5)	19(55.9)	
	Others	157(21.8)	41(26.1)	35(22.3)	81(51.6)	
Smoking (n=725)						
	Yes	54(7.4)	15(27.8)	10(18.5)	29(53.7)	0.87
	No	671(92.6)	172(25.6)	143(21.3)	356(53.1)	
Alcohol use (n=725)						
	Yes	103(14.2)	27(26.2)	23(22.3)	53(51.5)	0.93
	No	622(85.8)	160(25.7)	130(20.9)	332(53.4)	
Physical activity (n=725)						
	Yes	132(18.2)	30(22.7)	29(22.0)	73(55.3)	0.67
	No	593(81.8)	157(26.5)	124(20.9)	312(52.6)	
Planned pregnancy (n=716)						
	Yes	258(36.1)	74(28.7)	58(22.5)	126(48.8)	0.21
	No	458(63.9)	109(23.8)	95(20.7)	254(55.5)	

* Kruskal-Wallis test /

** Chi-square test /

a,b,c Dunn's post-hoc test; medians followed by different letters are significantly different from each other.

Note: GWG = gestational weight gain; IQR = interquartile range; HDP = hypertensive disorders of pregnancy; GDM = gestational diabetes mellitus; IOM = Institute of Medicine

Most of the women with insufficient GWG (54.5%) was classified as medium/high risk pregnancy, while low risk pregnancies were the majority for adequate (58.2%) and excessive (53.8%) groups (p=0.05). The IQR of the median number of consultations with a nutritionist was higher among the women with insufficient GWG (0–3) than it was among the women with adequate (0–2) and excessive (0–2; p=0.008) GWG. Among women with overweight (60%) and low pre-gestational weight (59.4%), we observed the highest proportions of excessive GWG (p<0.001). Among women with pre-gestational obesity, they had a lower proportion of excessive weight gain (48.3%) (Table 4).

The women with insufficient GWG had a higher median pregestational BMI (28.56 [24.34–32.2] kg/m²) than the women in the other categories (p<0.001). As for absolute GWG, those with insufficient GWG had the lowest median (3.7 [1.35–5.8] kg) and those with excessive GWG had the highest median (14 [11.32–17.64] kg; p<0.001). As for the adequacy of the women's GWG, it was found that excessive GWG was more prevalent than adequate or insufficient GWG for all four pregestational BMI categories. The highest proportion of adequate GWG was observed among the women with normal pregestational BMI (28.5%, p=0.001) (Table 4).

Turning to maternal complications, it was found that 64.4% of the women with HDP had excessive GWG and only 15.6% of those who developed this complication had insufficient GWG. Among the women diagnosed with GDM and HDP, 55.9% had excessive GWG (p<0.001) (Table 4).

When analyzing GWG according to the Brazilian charts,^(15)^ 25.8% (n=187) of the women were classified as having insufficient GWG, 21.1% (n=153) as having adequate GWG, and 53.1% (n=385) as having excessive GWG. When the IOM recommendations^(3)^ were used, 33.6% (n=244) of the women were classified as having insufficient GWG, 31.2% (n=226) as having adequate GWG, and 35.2% (n=255) as having excessive GWG. There was a significant association between the two GWG recommendations (p<0.001) (Table 3).

All the women classified as having insufficient GWG by the Brazilian charts were also classified as having insufficient GWG by the IOM recommendations. Among those who had adequate GWG according to the Brazilian charts, 37.2% (n=57) were classified as inadequate and 62.7% (n=96) as adequate by the IOM. Finally, among those with excessive GWG according to the Brazilian charts, 33.8% (n=130) were classified as having adequate GWG and 66.2% (n=255) as having excessive GWG according to the IOM recommendations.

The sensitivity and specificity of the Brazilian charts for the classification of insufficient GWG were 76.6 and 20, respectively; for the classification of excessive GWG, they were 100 and 72.3, respectively. The matrices used for these calculations are shown in table 5.

**Table 5:** Matrix for calculating the sensitivity and specificity of excessive and insufficient gestational weight gain according to the Brazilian charts, in relation to the IOM recommendations

		Adequacy of gestational weight gain according to the IOM ranges		Total
		Excessive / Insufficient	Adequate and insufficient / Adequate and excessive	
Adequacy of gestational weight gain according to the Brazilian charts^(15)^	Excessive / Insufficient	a	b	a+b
	Adequate and insufficient / Adequate and excessive	c	d	c+d
	Total	a+c	b+d	a+b+c+d

Indicators: Sensitivity (%) = a / (a+c) x 100; Specificity (%) = d / (b+d) x 100

## Discussion

Our study sample was composed of women with low- and medium/high-risk pregnancies, making it a pioneer in the analysis of the adequacy of GWG according to the Brazilian charts in a population that includes high-risk pregnancies, since the few published studies deal only with healthy pregnant women. In view of the scarcity of comparable data based on the Brazilian charts, most of the studies selected for this discussion used the IOM recommendation to classify the adequacy of GWG.

The median age at conception was higher for insufficient GWG than excessive GWG. A narrative review published in 2020 presented a causal modal of weight gain in young woman and concluded that younger ones have a poorer diet quality, which is associated with weight gain.^(18)^

Hermanussen and Scheffler,^(19)^ in an editorial, established an inverse relationship between GWG and parity. It was suggested that with the global growth in excessive GWG, postpartum weight retention is also increasing. Thus, the prevalence of overweight and obesity in women of childbearing age is rising, causing more women to start subsequent pregnancies overweight and consequently having lower GWG.^(19)^

In our study, gestational risk, which was classified by the level of prenatal care, was significantly associated with GWG. Despite the broad coverage of prenatal care in Brazil, studies have shown that its quality needs to improve, including early initiation and consultations with a nutritionist as part of primary care.^(20,21)^ The WHO regards prenatal nutritional care as an indicator of the quality of prenatal care and as contributing to a positive pregnancy experience.^(9)^

Our study also found that the number of consultations with a nutritionist was significantly associated with GWG, and that the women with insufficient GWG had more consultations with a nutritionist. In another study in Brazil, Padilha et al.^(22)^ found a higher prevalence of adequate GWG among women with a higher average number of prenatal consultations with a nutritionist, and that the women with high-risk pregnancies also had more consultations with a nutritionist. Therefore, our findings may indicate the need for more nutritional follow-up to achieve adequate GWG.^(22)^

The prevalence of women with low weight and normal weight in our study was comparable to that found in a European study, and the prevalence of women with overweight and obesity was comparable to that reported in the US.^(23,24)^ Pregestational BMI is considered a predictor of GWG, and we found a significant association between these two variables.^(25)^ According to Rouhana et al.,^(26)^ women with pregestational obesity have a higher risk of excessive GWG. However, in our study, the prevalence of excessive GWG was higher among the women classified as pregestational underweight and overweight, while those who began pregnancy with obesity had a lower prevalence of excessive GWG. This result suggests that the highest prevalence of insufficient GWG was among the women with obesity.

For normal-weight women, the risk of negative perinatal outcomes exists in cases of both insufficient and excessive GWG. However, studies suggest there is a higher risk of these outcomes, which include HDP, preterm birth, and LGA at birth, for women with pregestational obesity.^(2)^ It is estimated that 24% of any gestational complication can be attributed to maternal overweight and obesity. In addition, 32% of the occurrences of LGA newborns are attributed to excessive GWG.^(25)^

A meta-analysis carried out with more than one million women found that insufficient GWG was associated with gestational complications, as already established in guidelines.^(2,9,25)^ In our study, when analyzing maternal complications by GWG, the category of GWG with the highest proportion of women diagnosed with GDM was insufficient GWG, while excessive GWG predominated among the other complications. Overall, 80.8% of the women who had some complication had excessive GWG. These findings suggest that the incidence of maternal gestational complications could be reduced by controlling GWG.

The Brazilian Diabetes Society defines excessive GWG as a risk factor for the development of GDM.^(27)^ However, in our study, GDM was most prevalent among the women classified as having insufficient GWG, and more than half of those who did not have GDM had GWG above the recommended level. García-Moreno et al.,^(28)^ in a meta-analysis that aimed to describe the effect of constant glucose monitoring in women with GDM, found that those who constantly monitored their glucose tended to have lower GWG. The authors posited that this could be attributed both to greater care with diet, since dietary therapy is the first-choice therapeutic line for the condition, and to constant monitoring.^(27,28)^

Our study found that 57.5% of the participants with adequate GWG according to the IOM recommendations were classified with excessive GWG by the Brazilian charts, increasing the number of cases from 35.2% (IOM) to 53.1% (Brazil). On the other hand, the number of women classified with insufficient GWG was lower in the Brazilian classification, since 23.4% were designated as having adequate GWG. A study published in *The Lancet* suggests that GWG ranges lower than those recommended by the IOM would be beneficial for reducing levels of postpartum weight retention and thus visceral fat retention, thereby reducing mortality risk.^(29)^ Therefore, the Brazilian classification appears to be favorable in this regard, enabling intervention as soon as the inadequacy of GWG is identified, aiming to reduce undesirable maternal and neonatal outcomes and the risk of maternal mortality.

The sensitivity and specificity of the Brazilian GWG charts were calculated, considering the IOM recommendations as the standard test, since their validity for women with low- and high-risk pregnancies is well established in the literature.^(22,27)^ The classification of women with excessive GWG using the Brazilian charts was more consistent, identifying both real cases of excess GWG and avoiding errors in this classification, as it has maximum sensitivity and good specificity. For insufficient GWG, despite presenting good sensitivity, the specificity of the Brazilian charts was low. Therefore, the Brazilian charts can correctly identify most true cases of insufficient GWG; however, they may erroneously include pregnant women who do not have insufficient GWG in this category.

Our study was based on data collected between 2021 and 2023, when the Brazilian Ministry of Health recommended the IOM method for monitoring GWG adequacy.^(2,30)^ This may have increased the number of women classified as having excessive GWG, affecting adequacy of GWG according to the Brazilian charts. In addition, there are few published studies that analyze adequacy of GWG using the Brazilian charts, which makes it difficult to discuss and compare the findings of this study.

## Conclusion

The prevalence of each GWG classification range according to the Brazilian weight gain charts was 25.8% for insufficient, 21.1% for adequate, and 53.1% for excessive GWG. Compared to the IOM recommendations, the prevalence of insufficient and adequate GWG was lower and the prevalence of excessive GWG was higher. The sensitivity and specificity of the Brazilian charts were maximum and good, respectively, when classifying excessive GWG, and good and low, respectively, when classifying insufficient GWG. Among the factors associated with GWG, pregestational BMI and the number of consultations with a nutritionist can be modified. These findings reaffirm the national and international recommendations regarding the importance of controlling GWG during prenatal care. Research on gestational and neonatal outcomes in medium- and high-risk pregnant women, considering the recommendations of the Brazilian tables, is needed to consolidate their use in clinical practice.

## Data Availability

The authors did not make the data from this article available in repositories prior to submission.
